# Analysis of Trends in Demographic Distribution of Dental Workforce in the Kingdom of Saudi Arabia

**DOI:** 10.1155/2022/5321628

**Published:** 2022-10-19

**Authors:** Abdullah Saad Alqahtani, Nasser Raqe Alqhtani, Khalid Gufran, Ibrahim Saleh Aljulayfi, Abdulaziz M. Alateek, Soltan Ibrahim Alotni, Anas J. Aljarad, Albatool A. Alhamdi, Yasser Khaled Alotaibi

**Affiliations:** ^1^Department of Preventive Dental Sciences, College of Dentistry, Prince Sattam Bin Abdulaziz University, Alkharj, Saudi Arabia; ^2^Department of Oral and Maxillofacial Surgery and Diagnostic Science College of Dentistry, Prince Sattam Bin Abdulaziz University, Alkharj, Saudi Arabia; ^3^Department of Prosthetic Dental Sciences, College of Dentistry, Prince Sattam Bin Abdulaziz University, Alkharj, Saudi Arabia; ^4^College of Dentistry, Prince Sattam Bin Abdulaziz University, Alkharj, Saudi Arabia; ^5^College of Dentistry, King Saud University, Riyadh, Saudi Arabia; ^6^Prince Sultan Medical Military City, Riyadh, Saudi Arabia

## Abstract

Dental professionals are playing an imperative role in the healthcare system. It is important to distribute the dental workforce across the country. Therefore, this study aimed at analyzing the recent distribution of the dental workforce in the Kingdom of Saudi Arabia (KSA) and determining the current dentist-to-population ratio in the KSA. This is a cross-sectional study focused on the dental workforces working in the KSA between 2015 and 2020. Complete data of dentists working in the KSA with different professional ranks were obtained. The data were stratified by gender, professional rank (Saudi and non-Saudi), area of working (13 provinces in the KSA), and sector of working (public and private). A complete list of all dental universities was obtained to identify the increasing number of dental institutes at this current moment. In addition, the dentist-to-population ratio was also evaluated based on the current inhabitant in the KSA and the total dental surgeons. There are a total of 27181 dental surgeons and 8022 dental auxiliaries registered in different specialties as of 2020. Saudi citizens are holding the majority of the posts in both dentist and dental auxiliary categories. The percentage of males and female is slightly higher in dentists and dental auxiliaries, respectively. It also indicated that where most of the dental personnel work in the private sector, dental auxiliaries work in the public sector. Moreover, the highest number of dental workforces is identified in the Riyadh region among all the 13 provinces. Based on the databases, the current dentist-to-population ratio is 1 : 1288.16. In conclusion, the number of dental professionals is ample; however, rural areas lack specialists. Saudi dentists are progressively replacing foreign dentists in different professional ranks working in the KSA.

## 1. Background

Dental professionals fulfilled the need for oral health and the demand of society; therefore, it is imperative to maintain the dental workforce resources sufficient for the population of any country. There is a shortage of health workforce around the world including the dental workforce [1]. As the global population is increasing day by day, it would be ideal for every country to consistently inspect the need for a dental workforce, skills, and expectations along with the change in the population [2].

Oral health is an important part of overall health, which could be affected by several health conditions [3]. Any individual could not be deemed healthy without good oral health. Therefore, improved oral health is an important issue and mandatory to maintain among populations [4]. However, every country has substantial barriers to providing basic healthcare as well as oral healthcare to its population [5]. The insufficient number of trained healthcare providers, ignorance, and financial resources are the most common barriers in developing countries. Moreover, funds are not always properly allocated even in developed countries to assure access to healthcare especially dental healthcare [5]. A dental workforce study on Oman stated that an increasing number of Omani dental students are able to reduce the dentist-to-population ratio gradually since 2012 [6]. On the other hand, excessive numbers of dental students might worsen the socioeconomic situation, which leads to unemployment. For example, the rising number of dental institutes in Chile that graduate many students every year, which anticipated that there would be 77.5% surplus dentists by 2025 [7]. Systematic planning of establishing dental institutes is necessary to overcome the maldistribution of dentists [8]. Therefore, a dentist-to-population ratio is an important area to account and many countries around the world assessed this topic. Shanghai is one of the biggest cities in China exhibiting a 1 : 5201 dentist-to-population ratio, which is adequate for the inhabitants of Shanghai cities; however, dentists are equally distributed throughout the whole city and many suburbs lack dentists [9]. Likewise, in northern China, where the dentists working in rural areas are less knowledgeable and less specialized [10]. However, the national survey of China showed that there is a large gap between the original number of dental workforce and the demand for dental workforce, especially in rural areas [11]. Moreover, the Kentucky counties in the USA also showed similar disparities in the distribution of dental professionals throughout the counties [12]. Therefore, policymakers should distribute dental workforces appropriately throughout the country. A prediction study in Japan showed that if policymakers do not account for the distribution seriously, dental workforces lead to unequal allocation in the future [13]. Moreover, many countries are dependent on expatriates to fulfill the demand for healthcare in their nation. However, there are always sociocultural and language barriers between patients and foreign dentists. Countries such as Oman and Kuwait are taking necessary policies and attempts to replace foreign dentists with national dentists [6, 14]. However, based on the report, it will still take some longer time to completely replace the foreign dentists.

The Kingdom of Saudi Arabia (KSA) is the largest country in the Middle East consisting of about 35 million population according to https://www.stats.gov.sa/en/43. The Ministry of Health (MoH) and the public health system are the two main organizations that are providing different health services to the citizens free of cost since their establishment. The welfare policy of the KSA ensued improved population health among the other countries including the countries under Gulf Cooperation Council (GCC) [15]. The public sector offers free health services not only to Saudi citizens but also to expatriates living in the KSA [15–17]. The KSA has significantly upgraded its healthcare system over the previous few decades. The healthcare system is mainly split into public and private sectors. Ministry of health centers along with some agencies, such as ARAMCO health services, teaching hospitals, referral hospitals, national guard health affairs, and armed forces medical services are some of the examples of the efficient healthcare providers in KSA. Both private and public sectors provide healthcare at primary, secondary, and tertiary levels. Primary healthcare centers, hospitals, and general or specialized hospitals are affiliated with primary, secondary, and tertiary levels, respectively [18].

While the KSA is improving the healthcare system, the government of the country consistently prioritizes the healthcare sector by allotting an enormous budget annually [19, 20]. In 2019, 8.41% of the total national budget was allocated for healthcare, which is more than the budget of the previous 5 years [19]. However, the exact budget for oral healthcare is unknown. Even though a huge budget is allocated to healthcare, the government of the KSA still facing challenges to provide services to the nation due to the fast-growing population [20]. Therefore, the question arises: is the dental workforce adequate in the KSA with the increasing population? The growing number of populations in the KSA triggers the healthcare cost that encounters the maintenance of the proper healthcare system throughout the country. Moreover, local healthcare professionals are lacking in number; therefore, hiring foreign healthcare professionals are obligatory to provide healthcare services [18]. The satisfaction rate of patients is proportional to the healthcare services, and in 2020, the satisfaction rate of the patients was 72.75%. However, the different regions across the country showed different scores from 59.82 to 92.52% [19].

Oral healthcare is a significant part of overall healthcare. Although there is a positive development in the healthcare system across the country, dental caries significantly increased in the last few years [21–24]. This rising amount of caries rate among the citizens of KSA is indicating that the proper oral healthcare sector is still lacking in different regions [19, 25, 26]. However, consuming excessive sugar and soft drinks and not visiting the dentist might play an important role in poor oral health [27]. Dentist-to-population ratio is another issue that may impact overall oral healthcare [28].

Saudi Commission for Health Specialty (SCFHS) is the responsible authority that controls the health professions in the KSA including the dental profession as well as is accountable for registration, licensing, and recognition residency programs. The number of dental institutes significantly added in the last two decades resulting in a surge of dental graduates in the KSA. This increasing number is beneficial as it decreases the dentist-population ratio. The dentist-to-population ratio was 1 : 8906 in 1987 [29], which decreased to 1 : 1880 in 2016 [28]. The dentist-to-population rate in European countries ranges from 5.07 to 7.03 [30, 31]. Moreover, the highest and lowest dentist-to-population ratios are available in Japan (7.7) and China (0.12), respectively [32].

In order to maintain the international rank, it is imperative to evaluate demographic distribution in every field of the healthcare sector from time to time. Very few studies have been performed in the field of dentistry to identify the status of dental healthcare in the KSA. The last study was published in 2016. Some authors have updated the regional distribution of dentists recently [19, 33]; however, a distribution for the whole country is missing at this moment. Based on the previous studies conducted in the KSA and the efforts government is delivering to the healthcare system, the total number of dental workforces should increase along with the increasing population, which ultimately reduces the dentist-to-population ratio compared to the previous studies. Moreover, to achieve the goal of the “Vision 2030” policy in the KSA, more Saudi professionals should replace non-Saudi professionals. As per the literature search, no recent data have exhibited the recent status of the dental workforce in the KSA and the current ratio of dentist-to-population in the KSA for the last few years. Therefore, this descriptive cross-sectional study aimed to identify whether the dental workforce is adequate to serve the people in the KSA, the recent distribution of the dental workforce, and ascertain the current dentist-to-population ratio. Moreover, it also indicates the future direction of the distribution of dentists in the KSA to deliver the best dental care throughout the nation.

## 2. Materials and Methods

This cross-sectional study focused on the dental workforces working in the KSA between 2015 and 2020. All the data were collected from the SCFHS by approaching officially through a request from the College of Dentistry, Prince Sattam Bin Abdulaziz University. Complete data of dental professionals were obtained, which included a total number of dentists in different specialties, professional ranks, and dental auxiliaries working in the KSA from 2015 to 2020. The data were further stratified with gender, professional rank (Saudi and non-Saudi), area of working (13 provinces in the KSA), and sector of working (public and private). The dental workforce was divided into 17 specialties based on their license approved by the SCFHS, and all the data were scrutinized based on these specialties. Moreover, along with the dental workforces, the list of all dental universities and colleges (private and public) was obtained to identify the rising number of dental institutes at the current period in the KSA. In addition, the dentist-to-population ratio was also assessed based on the current inhabitant in KSA and the total dental surgeons actively working in the KSA. This study was approved by the Ethical Committee in the Health and Science Discipline (REC-HSD) of Prince Sattam Bin Abdulaziz University with approval no. REC-HSD-102-2021.

Descriptive statistics including frequency and percentages were calculated to characterize the gender, working sector, working region, and professional rank of the dentists and dental auxiliaries. The chi-square (*x*^*2*^) test was used to investigate the distribution of dentists and dental auxiliaries based on their professional rank, gender, work sector, and workplaces. All the analyses were performed in the spreadsheets from Microsoft Excel 2021 (Office 365, Microsoft Corporation).

## 3. Results

There are a total of 27181 dental surgeons and 8022 dental auxiliaries licensed in the SCFHS in different specialties as of 2020. The distribution of the recent SCFHS data is presented in Table 1 which showed that there are 54.80% Saudi and 42.20% non-Saudi citizen dental employees working in different professional ranks in the KSA. Along with that, the percentage of males (54.86%) is slightly higher than their female (45.14%) counterparts. It also showed that more than half of the total dental personnel work in the private sector (66.15%) compared to the public sector (33.85%). Moreover, the highest numbers of dentists are identified in the Riyadh region (37.72%) among all the 13 provinces followed by the Makkah region (23.51%).  Table 2 represents the distribution of dental auxiliaries including dental assistants, dental hygienists, and dental technicians. It showed that 60.98% of total dental aids are female and the rest are male. Saudi nationals (72.65%) are holding more posts than non-Saudi citizens (27.35%). Moreover, 64.40% of dental auxiliaries are working in the public sector and only 35.60% are working in the private sector. Likewise, dental practitioners and most of the dental auxiliaries work in the Riyadh region (40.54%).

**Figure 1:**
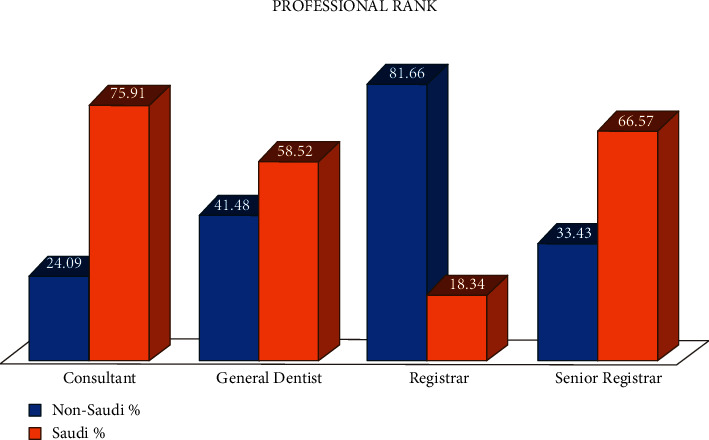
Distribution of Saudi and non-Saudi dentists in different professional ranks.

Dental surgeons are working under four professional ranks, such as consultant, senior registrar, registrar, and general dentist.  Figure 1 shows that Saudi citizens are higher in numbers in all ranks compared to non-Saudi citizens except for the registrar rank. The percentage of Saudi dentists is higher in consultant rank followed by the senior registrar position. There is a significant difference (*P* = 0.0001) between the Saudi and non-Saudi nationals working in different professional ranks.

The whole dental workforce is comprised of different specialties, and Table 3 shows that a total of 16936 and 8668 dental personnel work in the private sector and public sector, respectively. However, most dental specialists mostly work in the public sector. The private sector is mostly surpassed by general dentists. Likewise, Table 4 exhibits that a total of 5166 and 2856 dental auxiliaries are working in the public and private sectors, respectively. There is a significant difference (*P* = 0.0001) observed between the private and public sectors across the KSA where dentists and dental auxiliaries work in different specialties.

From the overall distribution, 2641 additional male dentists are working in the KSA who are licensed with the SCFHS. However, in a few specialties, such as dental public health, oral medicine, oral pathology, and paediatric dentistry, female dentists are more than male dentists (Table 5). In addition, the total number of male dental auxiliaries is fewer (39.02%) than that of their female counterparts (60.98%) (Table 6). There is a significant difference (*P* = 0.0001) observed between the gender among dentists and dental auxiliaries who are working in different specialties.

The distribution of the dentists and dental auxiliaries in all 13 provinces of the KSA is presented in Tables 7 and 8 which exhibited that the most dental workforces are working in Riyadh and the least numbers of dental workforces are working in Al Bahah. Moreover, some of the regions lack different specialties; that is, only 30 dental implantologists work around the KSA and 11 implantologists among them work in the Riyadh region. Regions, for example, Al Bahah, Al Jawf, Jizan, Najran, and the northern border do not have any implantologist in both the private and public sectors. This scenario is similar for other specialties too. There is a significant difference (*P* = 0.0001) between dentists and dental auxiliaries working in different regions of the KSA.

**Figure 2:**
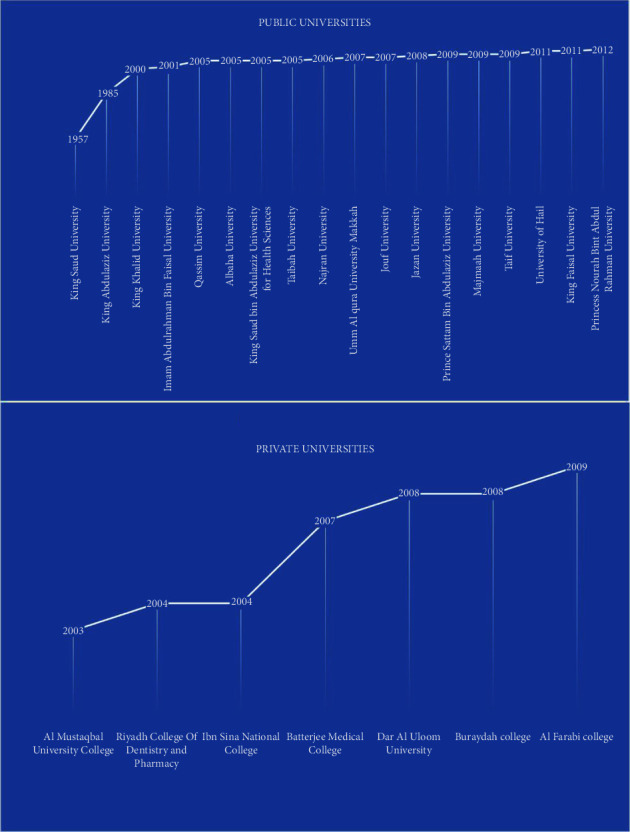
Public and private universities in the KSA.

There are a total of 18 public and seven private universities and colleges established in the KSA, which are offering dentistry programs since 1957. Although only two universities were offering a dentistry program until 1999, the rest of the universities and colleges were rapidly instituted from 2000 to 2012.  Figure 2 shows the growth of public and private dental universities and colleges till 2020. The last dental university was established in 2012.

As per the government statistics department, there are a total of 35013414 people residing in the KSA and the record of the SCFHS showed 27181 licensed dentists working till 2020. Therefore, there was a 1 : 1288.16 dentist-to-population ratio available until 2020 in the KSA.

## 4. Discussion

This current study exhibited the demographic distribution of dental workforces from 2015 to 2020 in the KSA. The outcome of this study showed that a total of 27,181 dentists are available in the KSA in different professional ranks. This total number of dentists indicates the proliferation of dentists since 2016. AlBaker et al. examined the demographic distribution of dentists until 2016 and found 16,887 dentists working across the KSA [28]. In less than five years, the dentist workforce turns out to be near to double in numbers. However, there was a limitation in attaining the current data as many important variables could not be compared. For example, AlBaker et al. compared the overall distribution of age, work region, gender, professional rank, and sector between the Saudi and non-Saudi populations. However, the current study could compare only the professional ranks of the national and foreign workforce [28].

Among all the dentists, 54.80% were Saudi citizens and 42.20% were foreign dentists working in different professional ranks until 2020, which denotes an immense growth in Saudi dentists. Until 2016, there were only 22.09% of Saudi dentists working in the KSA, and in the last four years, it enhanced more than twice in number. These statistics showed that, although there were shortages and a high unemployment rate among Saudi dentists, higher authorities are succeeding to close the gap between Saudi and non-Saudi dentists.

Among all the dental surgeons, general dentists are more prevalent (74.48%) in the KSA than those in the other professional ranks. Only 25.52% of specialists in different branches of dental science are working in the KSA. Other than the general dentists, the Saudi Board of Prosthodontists (4.59%) and Endodontists (3.52%) are the two most prevalent specialties found in the KSA. A total of 54.72% of prosthodontists work in the private sector. However, more than half of the endodontists (58.82%) work in the public sector. On the other hand, dental implantologists (0.12%) and oral and maxillofacial radiologists (0.17%) are the two least specialties work in the KSA. Whereas most of the dental implantologists work in the private sector (70.97%), oral and maxillofacial radiologists work in the public sector (81.82%). Males are more prevalent in all these four specialties. The small percentages explain that there are still opportunities for implantologists and radiologists to seek vacancies in both public and private sectors in the KSA. These results also reflect in the different regions of the KSA. The Saudi Board of Prosthodontics and the Saudi Board of Oral and Maxillofacial Surgeons are more prevalent in most of the regions along with endodontists. Moreover, along with dental implants and radiology, a few other specialties are also absent in some regions. There is a lack of dental public health specialties in the Al Jawf and Tabuk regions, which is an important specialty in Dentistry. Moreover, dental public health practitioners are very less (0.41%) in the overall KSA. The scarcity of dental health practitioners may adversely affect the community health system in the whole country. This might be the possible reason for increasing the caries rate around the country, which was mentioned in the previous studies conducted in the KSA [19]. In addition, advanced general dentistry and oral pathology in Al Jawf, conservative dentistry, oral medicine and oral pathology in Hail and the northern border, family dentistry in Jizan, and oral medicine specialties are missing in the Tabuk region. In order to provide optimum oral healthcare, these specialties need to be filled in all the regions in the KSA.

The percentage of Saudi and non-Saudi general dentists among all dental surgeons are 43.59% and 30.89%, respectively. The percentage of both Saudi and foreign general dentists in 2020 decreased compared to 2016 [28]. In recent years, most of the posts for general dentists were filled by Saudi citizens and many specialists' positions were also occupied by Saudi citizens, which were unlike a few years back. The registrar rank is only surpassed by the non-Saudi specialists compared to the Saudi dentists at the current moment. It is believed that the increased number of Saudi residency programs is playing important role in the invasion of Saudi dentists in many specialists' positions compared to the previous years. This comparison is important in terms of the perspective of vision 2030. As the KSA intends to replenish the maximum healthcare sector with national professionals, this distribution could provide insight into which professional rank lacks the national dental force. Moreover, dentists and dental auxiliaries from many developing southeast Asian countries and African countries are continuously coming to work in the KSA due to the better salary structure and healthier living conditions. Hence, the recent statistics of the foreigners and national dental workforce would enlighten the foreign nationals on how long and which professional rank would be welcomed by the KSA to work in the future.

Gender distribution is considered to be an imperative factor in any dental workforce around the world. The female dentists (45.14%) are slightly fewer than the male dentists (54.86%) in the KSA and the proportion is distinct from 2016 when female and male dentists were 38.94% and 61.06%, respectively [28]. Thus, comparing the previous report, it could be stated that the number of female dentists is progressively increasing in the field of dentistry in the KSA. In addition, the number of female specialists increased almost in double since last four years. According to AlBaker et al., there were 1402 female specialists, which increased to 2494 in this study [28]. Yet, male dentists have slightly increased in the number overall ratios. The proportion of male and female dentists is also likewise in comparison with the other counties. Most countries showed a lower proportion of female dentists working in the dental workforce than their counterparts [32, 34–36].

This demographic statistic showed that most of the dentists are working in the private sector, with only one-third of the total dentists working in the public sector. According to AlBaker et al., the statistics were similar until 2016; therefore, it could be predicted that not many positions for dentists increased in the public sector. However, a previous study classified Saudi and non-Saudi citizens in different sectors, which exhibited that two-thirds of Saudi dentists work in the public sector and most of the foreign dentists work in the private sector [28]. Nevertheless, this study could not classify that due to the lack of appropriate data. Statistics from densely populated countries like India indicated that only 5% of dentists work in the public sector, which is way too less in number than the KSA [37]. On the other hand, there were only 1% of Omani dentists worked in the private sector [6]. In addition, although the demand for the private sector increases day by day, the preponderance of dentists is working in the public sector in Malaysia [38].

The working area is also an important factor to provide healthcare to the general population. The KSA is the largest country in the Middle East consisting of 13 provinces. The outcome of this study presented that a supreme number of dentists work in Riyadh, which is the capital of the KSA. This result is similar to the finding of AlBaker et al. [28]. The second-highest numbers of dentists work in the Makkah region, and the least number of dental surgeons work in Al Bahah. As Makkah is the third largest province and Al Bahah is the smallest province in the KSA, the above fact is justified. Although most of the dentists are employed in Riyadh, a study on private dental clinics in Riyadh showed that most of the clinics are situated in Olaya and Rawdha municipalities. Moreover, it also stated that private clinics are mainly operated by non-Saudi male dentists, many areas inside Riyadh city lacks dental clinics [39]. Similar reports were found in a recent publication based on Al Ahsa private clinics [33]. Al Ahsa private clinics are mostly operated by Egyptian dentists. Although Saudi general dentists are joining private clinics in Al Ahsa for the last three years, specialists are still lacking [33]. In addition, it was also noted that fresh dental graduates are reluctant to work in the rural areas of Riyadh; therefore, there is still a shortage of dentists in many parts of Riyadh [40]. This current study identified that many specialties are missing in many provinces, such as there being no dental implant specialists in Al Bahah, Al Jouf, Jizan, Najran, and Northern Province. There was only one dental implant specialist in the Qassim and Asir regions. So, it could be assumed that oral health services are compromised in many of the provinces except the major provinces. It is important to present a minimum number of specialists from all specialty groups in each province. This unequal distribution is a concern not only for the KSA but also for other countries around the world.

This study presented that for dental auxiliaries, females are higher in number than males, which is the reverse scenario of dentists working in the KSA. However, the number of males is higher in dental technician and dental hygienist posts. Most of the dental assistant or dental nurse position is filled with female workers. Although non-Saudi citizens are slightly higher in numbers in dental technician posts, Saudi citizens are holding most of the dental auxiliary positions in the KSA. Moreover, more than 95% of the total dental hygienists are Saudi nationals. Unlike dentists, dental ancillaries mostly work in the public sector. Only the number of dental technicians is higher in the private sector. Therefore, it could be stated that as non-Saudi citizens are greater in dental technician position, job opportunity is more in the private sector for this position or vice versa. Due to the presence of most dental hospitals and clinics in the Riyadh region, most of the dental ancillaries are working in this region. The second-highest region is Makkah for dental professionals; however, the Eastern province is the second-highest region for the orting dental staff. Moreover, unlike dentists, a least number of dental auxiliaries are working in the Al Bahah region. The finding of the dental ancillaries from this study could not be compared with any previous studies as no study has been found that assessed the distribution of dental auxiliaries in the KSA previously.

The dentist-to-population ratio is the key to indicating the acceptable number of oral healthcare specialists compared to the total population. This ratio is decreasing in the KSA with time. In 1987, the ratio was 1 : 8906 [29], which decreased in 2016 to 1 : 1880 [28]. The current study revealed that the ratio further decreased in 2020 to 1 : 1288.16 or 7.7 dentists per 10,000 population. In 2013, there were 2.3 dentists per 10,000 population [6] in the KSA; hence, the improvement in the ratio is commendable. The number of dentists is meticulously related to the number of graduates who attain dental degrees each year. Currently, there are a total of 18 public and seven private dental colleges present in the KSA. There were many dental colleges established in the KSA after the year 2000; therefore, the dentist-to-population ratio decreased immensely, which is considered beneficial for the general population. However, most of the universities are situated in the Riyadh and Makkah regions. Other regions are still lacking dental colleges. Establishing dental colleges across all the provinces not only improves the healthcare system but also opens many positions for dental specialists to serve the nation. Of the total dentists working in the KSA, 74.48% were general dentists. The number of specialties still needs to be enriched for better healthcare services. Therefore, different postgraduation programs need to initiate miniating the general dentists and specialist's ratio. Currently, there are insufficient opportunities for Saudi citizens or residents to attain postgraduation training in the KSA. Although there are a total of 25 dental colleges available in the KSA, not all of them have postgraduation training programs. If the dental colleges were properly distributed to all 13 provinces equally with the postgraduation opportunities in different fields of dentistry, the general dentists and specialist's ratio would improve immensely. In addition, postgraduation training requires clinical experience with real patients; consequently, more residents in the specific region would receive quality treatment conveniently. The oral healthcare system is already in a better position based on the dentist-to-population ratio compared to the other countries; it would improve enormously without any doubt if the postgraduation system expands purposefully.

Improving the healthcare system is one of the main areas of Vision 2030. According to the highlight of vision 2030, the private sector would play an important role in economic growth; therefore, the privatization of healthcare services and expanding the resourceful utilization of accessible sources is one of the goals [33]. Consequently, the private sector requires to simulate responsibilities. As per previous studies, the private sector is growing fast with an assuring future; following Vision 2030, better work conditions and job security should be retained to inspire Saudi citizens to work in different private sectors. Moreover, it is also important to strategize the number of dental graduates from both private and public dental colleges for better employment.

This current data provides a huge insight into the dental workforce in the KSA. Although a few parts of the complete data are missing in this current project, it still could reflect the overall situation of dental workforces at the current stage. Health authorities could take necessary actions based on this current report such as increasing the number of different specialties in regions other than Riyadh and Makkah. The missing specialties need to be filled to provide optimal oral healthcare to the residents in rural regions. Moreover, fresh graduates from the different Saudi universities may decrease the population-to-dentists ratio, and graduates need to be encouraged to work in the periphery areas, which could improve the improper distribution of oral healthcare providers across the KSA. Moreover, the result exhibited that the Saudi Board of Prosthodontics and Saudi Board of Oral and Maxillofacial Surgery graduates are dominantly working in most of the region; therefore, more Saudi board postgraduation programs should be conducted to increase the specialists in the KSA.

### 4.1. Strengths and Limitations

Although there are limited data available related to the dental workforce in the KSA, this study presented all the major variables in dental workforces. Some of the parts could not be addressed due to the insufficient data provided by the SCFHS, such as specific subdivisions of gender and nationality of dentists and dental auxiliaries in different sectors, and the specific subdivision of gender and nationality in different regions. As per our best knowledge, this is the first study in the KSA, which included the complete dental workforce including dental auxiliaries along with dentists in different ranks. The previous studies only focused on dentists. However, further studies including the complete data of different variables are recommended.

## 5. Conclusions and Future Directions

The outcome of the current study exhibited the following:

1. The number of dental professionals is adequate yet unbalanced distribution around the whole country. Although the distribution of dental workforces improved over the last five years, fewer specialists are employed in the rural areas, which ultimately restricts the delivery of better oral healthcare to the people residing in peripheral areas.
2. Saudi dentists have replaced non-Saudi dentists in all professional ranks except the registrar rank. In addition, Saudi dental auxiliaries are also gradually replacing non-Saudi dental auxiliaries.
3. The results of this study may aid in the suitable distribution of dental professionals across the KSA. As the people who live in rural areas are not receiving oral healthcare due to the lack of professionals, oral health should be compromised in those areas. Few specialties such as the dental implant, radiology, dental public health, oral pathology, and oral medicine are less in numbers across the KSA and nil in some regions.
4. The private sector accommodates more dentists than the public sector. However, it is vice versa in the case of dental auxiliaries.
5. Male dentists are more prevalently working in the KSA, whereas females are more in numbers among dental auxiliaries.

Future studies on dental health in rural areas need to be conducted. Moreover, the regional-based study based on the distribution of dental professionals and the dentist-to-population ratio is required to identify to optimize the oral health workforce strengthening to meet population oral healthcare needs more equitably and/or efficiently. In addition, the geographic distribution of dental workforces and the location of their practice may inform the SCFHS and reimbursement of the dental workforce policies for attaining the target of Vision 2030 in the healthcare system.

## Figures and Tables

**Table 1:** The characteristics and distribution of dentists in the KSA.

Variable	Total	Percentage
Gender		
Male	14911	54.86
Female	12270	45.14

Nationality		
Saudi	14878	54.80
Non-Saudi	12274	42.20

Sector		
Private	16936	66.15
Public	8668	33.85

Region		
Al Bahah	143	0.58
Al Jouf	328	1.33
Al Madinah	1378	5.60
Al Qassim	1034	4.20
Asir	1317	5.35
Eastern Province	3252	13.22
Hail	474	1.93
Jizan	625	2.54
Makkah	5781	23.51
Najran	370	1.50
Northern borders	179	0.73
Riyadh	9277	37.72
Tabuk	436	1.77

Total (N)	27181	

**Table 2:** The characteristics and distribution of dental auxiliaries in the KSA.

Variable	Total	Percentage
Gender		
Male	3584	39.02
Female	5602	60.98

Nationality		
Saudi	6674	72.65
Non-Saudi	2512	27.35

Sector		
Private	2856	35.60
Public	5166	64.40

Region		
Al Bahah	78.00	0.95
Al Jouf	66.00	0.81
Al Madinah	452.00	5.53
Al Qassim	278.00	3.40
Asir	404.00	4.94
Eastern Province	1529.00	18.70
Hail	79.00	0.97
Jizan	164.00	2.01
Makkah	1367.00	16.72
Najran	140.00	1.71
Northern borders	63.00	0.77
Riyadh	3315.00	40.54
Tabuk	243.00	2.97

Total (N)	36367	

**Table 3:** The distribution of dentists in different sectors is based on specialties.

Specialty	Private	Public	*P* value
	N (%)	N (%)	
Advanced general dentistry	58 (25.11)	166 (71.86)	0.0001^*∗*^
Conservative dentistry	129 (51.39)	118 (47.01)	
Dental implant	22 (70.97)	9 (29.03)	
Dental public health	36 (33.33)	70 (64.81)	
Endodontics	371 (39.68)	530 (56.68)	
Family dentistry	94 (59.87)	42 (26.75)	
General dentistry	13743 (67.89)	5182 (25.60)	
Oral and maxillofacial radiology	8 (17.78)	36 (80.00)	
Oral medicine	23 (34.33)	39 (58.21)	
Oral pathology	14 (20.00)	49 (70.00)	
Oral surgery	166 (62.64)	96 (36.23)	
Orthodontics	411 (68.73)	162 (27.09)	
Paediatric dentistry	325 (40.63)	443 (55.38)	
Periodontics	269 (44.54)	312 (51.66)	
Restorative dentistry	188 (24.64)	536 (70.25)	
Saudi board of oral and maxillofacial surgery	436 (54.43)	346 (43.20)	
Saudi board of prosthodontics	643 (53.05)	532 (43.89)	
Total	**16936**	**8668**	

N: total number; %: percentage; ^*∗*^significant difference (<0.05) in the chi-square test.

**Table 4:** The distribution of dental auxiliaries in different sectors based on specialties.

Specialty	Private	Public	*P* value
	N (%)	N (%)	
Dental technician	1254 (53.41)	1094 (46.59)	0.0001^*∗*^
Dental hygienist	325 (28.04)	834 (71.96)	
Dental assistant	1277 (28.28)	3238 (71.72)	
Total	**2856**	**5166**	

N: total number; %: percentage; ^*∗*^significant difference (<0.05) in the chi-square test.

**Table 5:** The distribution of dentists by gender.

Specialty	Female	Male	*P* value
	N (%)	N (%)	
Advanced general dentistry	70 (30.30)	161 (69.70)	0.0001^*∗*^
Conservative dentistry	93 (37.05)	158 (62.95)	
Dental implant	3 (9.68)	28 (90.32)	
Dental public health	63 (58.33)	45 (41.67)	
Endodontics	330 (35.29)	605 (64.71)	
Family dentistry	46 (29.30)	111 (70.70)	
General dentistry	9776 (48.29)	10467 (51.71)	
Oral and maxillofacial radiology	22 (48.89)	23 (51.11)	
Oral medicine	38 (56.72)	29 (43.28)	
Oral pathology	37 (52.86)	33 (47.14)	
Oral surgery	60 (22.64)	205 (77.36)	
Orthodontics	233 (38.96)	365 (61.04)	
Paediatric dentistry	449 (56.13)	351 (43.88)	
Periodontics	222 (36.75)	382 (63.25)	
Restorative dentistry	348 (45.61)	415 (54.39)	
Saudi board of oral and maxillofacial surgery	117 (14.61)	684 (85.39)	
Saudi board of prosthodontics	363 (29.95)	849 (70.05)	
Total	**12270**	**14911**	

N: total number; %: percentage; ^*∗*^significant difference (<0.05) in the chi-square test.

**Table 6:** The distribution of dental auxiliaries by gender.

Specialty	Female	Male	*P* value
	N (%)	N (%)	
Dental technician	281 (11.52)	2158 (88.48)	0.0001^*∗*^
Dental hygienist	604 (48.40)	644 (51.60)	
Dental assistant	4717 (85.78)	782 (14.22)	
Total	**5602**	**3584**	

N: total number; %: percentage; ^*∗*^Significant difference (<0.05) in the chi-square test.

**Table 7:** The distribution of dentists by the region of Saudi Arabia.

Specialty	Al Bahah	Al Jawf	Al Madinah	Al Qassim	Asir	Eastern Province	Hail	Jizan	Makkah	Najran	Northern borders	Riyadh	Tabuk	*P* value
	N (%)	N (%)	N (%)	N (%)	N (%)	N (%)	N (%)	N (%)	N (%)	N (%)	N (%)	N (%)	N (%)	
Advanced general dentistry	2 (0.87)	0 (0)	2 (0.87)	3 (1.30)	14 (6.06)	22 (9.52)	1 (0.43)	3 (1.30)	18 (7.79)	1 (0.43)	0 (0)	159 (68.83)	2 (0.87)	0.0001^*∗*^
Conservative dentistry	1 (0.40)	7 (2.79)	14 (5.58)	12 (4.78)	14 (5.58)	28 (11.16)	0 (0)	9 (3.59)	64 (25.50)	4 (1.59)	2 (0.80)	70 (27.89)	6 (2.39)	
Dental implant	0 (0)	0 (0)	2 (6.45)	1 (3.23)	1 (3.23)	2 (6.45)	3 (9.68)	0 (0)	9 (29.03)	0 (0)	0 (0)	11 (35.48)	1 (3.23)	
Dental public health	1 (0.93)	0 (0)	10 (9.26)	4 (3.70)	3 (2.78)	9 (8.33)	2 (1.85)	3 (2.78)	19 (17.59)	1 (0.93)	2 (1.85)	47 (43.52)	0 (0)	
Endodontics	7 (0.75)	10 (1.07)	40 (4.28)	36 (3.85)	40 (4.28)	96 (10.27)	16 (1.71)	17 (1.82)	229 (24.49)	7 (0.75)	6 (0.64)	353 (37.75)	12 (1.28)	
Family dentistry	0 (0)	2 (1.27)	10 (6.37)	5 (3.18)	10 (6.37)	19 (12.10)	1 (0.64)	0 (0)	17 (10.83)	1 (0.64)	1 (0.64)	57 (36.31)	5 (3.18)	
General dentistry	102 (0.50)	251 (1.24)	1018 (5.03)	752 (3.71)	1004 (4.96)	2438 (12.04)	386 (1.91)	468 (2.31)	4128 (20.39)	281 (1.39)	141 (0.70)	6845 (33.81)	333 (1.65)	
Oral and maxillofacial radiology	0 (0)	1 (2.22)	2 (4.44)	0 (0)	2 (4.44)	4 (8.89)	0 (0)	4 (8.89)	12 (26.67)	0 (0)	0 (0)	18 (40.00)	0 (0)	
Oral medicine	1 (1.49)	1 (1.49)	3 (4.48)	3 (4.48)	1 (1.49)	7 (10.45)	0 (0)	3 (4.48)	19 (28.36)	1 (1.49)	0 (0)	17 (25.37)	0 (0)	
Oral pathology	3 (4.29)	0 (0)	4 (5.71)	2 (2.86)	1 (1.43)	6 (8.57)	0 (0)	4 (5.71)	17 (24.29)	1 (1.43)	0 (0)	19 (27.14)	1 (1.43)	
Oral surgery	2 (0.75)	6 (2.26)	24 (9.06)	15 (5.66)	15 (5.66)	46 (17.36)	5 (1.89)	6 (2.26)	69 (26.04)	3 (1.13)	6 (2.26)	49 (18.49)	6 (2.26)	
Orthodontics	3 (0.50)	5 (0.84)	29 (4.85)	29 (4.85)	26 (4.35)	65 (10.87)	11 (1.84)	19 (3.18)	144 (24.08)	13 (2.17)	7 (1.17)	162 (27.09)	15 (2.51)	
Paediatric dentistry	4 (0.50)	7 (0.88)	36 (4.50)	34 (4.25)	19 (2.38)	94 (11.75)	7 (0.88)	12 (1.50)	221 (27.63)	10 (1.25)	2 (0.25)	282 (35.25)	12 (1.50)	
Periodontics	2 (0.33)	6 (0.99)	35 (5.79)	27 (4.47)	30 (4.97)	74 (12.25)	5 (0.83)	15 (2.48)	158 (26.16)	6 (0.99)	3 (0.50)	203 (33.61)	7 (1.16)	
Restorative dentistry	2 (0.26)	3 (0.39)	32 (4.19)	15 (1.97)	41 (5.37)	69 (9.04)	9 (1.18)	9 (1.18)	184 (24.12)	6 (0.79)	1 (0.13)	344 (45.09)	5 (0.66)	
Saudi board of oral and maxillofacial surgery	7 (0.87)	16 (2.00)	41 (5.12)	49 (6.12)	36 (4.49)	113 (14.11)	13 (1.62)	25 (3.12)	182 (22.72)	18 (2.25)	2 (0.25)	233 (29.09)	15 (1.87)	
Saudi board of prosthodontics	6 (0.50)	13 (1.07)	76 (6.27)	47 (3.88)	60 (4.95)	160 (13.20)	15 (1.24)	28 (2.31)	291 (24.01)	17 (1.40)	6 (0.50)	408 (33.66)	16 (1.32)	
Total	143	328	1378	1034	1317	3252	474	625	5781	370	179	9277	436	

N: total number; %: percentage, ^*∗*^significant difference (<0.05) in the chi-square test.

**Table 8:** The distribution of dental auxiliaries by the region of Saudi Arabia.

Specialty	Al Bahah	Al Jouf	Al Madinah	Al Qassim	Asir	Eastern Province	Hail	Jizan	Makkah	Najran	Northern borders	Riyadh	Tabuk	*P* value
	N (%)	N (%)	N (%)	N (%)	N (%)	N (%)	N (%)	N (%)	N (%)	N (%)	N (%)	N (%)	N (%)	
Dental technician	9 (0.39)	42 (1.80)	206 (8.81)	114 (4.88)	171 (7.32)	316 (13.52)	38 (1.63)	45 (1.93)	407 (17.42)	49 (2.10)	24 (1.03)	851 (36.41)	65 (2.78)	0.0001^*∗*^
Dental hygienist	53 (4.52)	6 (0.51)	30 (2.56)	42 (3.58)	48 (4.10)	230 (19.62)	3 (0.26)	7 (0.60)	152 (12.97)	23 (1.96)	7 (0.60)	547 (46.67)	24 (2.05)
Dental assistant	16 (0.34)	18 (0.39)	216 (4.63)	122 (2.61)	185 (3.96)	983 (21.05)	38 (0.81)	112 (2.40)	808 (17.31)	68 (1.46)	32 (0.69)	1917 (41.06)	154 (3.30)
Total	78	66	452	278	404	1529	79	164	1367	140	63	3315	243

N: total number; %: percentage; ^*∗*^significant difference (<0.05) in the chi-square test.

## Data Availability

The data that support the findings of this study are available from the corresponding author, upon reasonable request.
